# Sensing in Inland Waters to Promote Safe Navigation: A Case Study in the Aveiro’s Lagoon

**DOI:** 10.3390/s24237677

**Published:** 2024-11-30

**Authors:** Diogo Miguel Carvalho, João Miguel Dias, Jorge Ferraz de Abreu

**Affiliations:** 1 DIGIMEDIA—Digital Media and Interaction Research Centre, Department of Communication and Arts, University of Aveiro, 3810-193 Aveiro, Portugal; diogocarvalho28@ua.pt; 2 CESAM—Centre for Environmental and Marine Studies (CESAM), Physics Department, University of Aveiro, 3810-193 Aveiro, Portugal; joao.dias@ua.pt

**Keywords:** internet of things, IoT systems, maritime data, meteorologic data, maritime communities, safe navigation, Raspberry Pi, ESP8266

## Abstract

Maritime navigation safety relies on preventing accidents, such as collisions and groundings. However, several factors can exacerbate these risks, including inexistent or inadequate buoyage systems and nautical charts with outdated bathymetry. The International Hydrographic Organization (IHO) highlights high costs and traditional methods as obstacles to updating bathymetric information, impacting both safety and socio-economic factors. Navigation in inland and coastal waters is particularly complex due to the presence of shallow intertidal zones that are not signaled, where navigation depends on tidal height, vessel draw, and local knowledge. To address this, recreational vessels can use electronic maritime sensors to share critical data with nearby vessels. This article introduces a low-cost maritime data sharing system using IoT technologies for both inland (e.g., Ria de Aveiro) and coastal waters. The system enables the collection and sharing of meteorological and oceanographic data, including depth, tide height, wind direction, and speed. Using a case study in the Ria de Aveiro lagoon, known for its navigational difficulties, the system was developed with a Contextual Design approach focusing on sailors’ needs. It allows for the real-time sharing of data, helping vessels to anticipate maneuvers for safer navigation. The results demonstrate the system’s potential to improve maritime safety in both inland and coastal areas.

## 1. Introduction

Maritime navigation is a combination of science and art, in which sailors must observe, understand, and analyze several factors that may affect navigation to ensure the vessel’s safety. Recording data and controlling a route to move a vessel from one location to another [1,2,3] is heavily dependent on a human’s skilled interpretation and the accuracy of the data collected by navigational instruments, including plotters, radars, and depth sounder transducers, to prevent conflicts and manage the vessels’ autonomy [1,4,5,6].

The importance of reliable navigational data becomes evident in different water environments [1], including ocean, coastal, and inland waters. In ocean and coastal navigation, vessels frequently face the challenge of entering inland or restricted waters [7,8]. Navigating in such waters can be difficult, and it is crucial to approach this task with caution and precision. Therefore, nautical charts are essential to determine the position, course, and route to be taken. Modern tools, such as chart plotters, have largely replaced traditional methods, providing real-time data that enhance navigational accuracy and safety. Although these modern solutions have an improved accuracy, they often fail to address the real-time data needed for safe navigation in inland and coastal waters. Notably, during this research, which began in 2021, PredictWind’s DataHub [9] emerged, offering real-time weather data for sailors. While promising, it is primarily focused on weather prediction and it requires expensive equipment and a subscription service, which can be unaffordable for many users.

**Figure 1:**
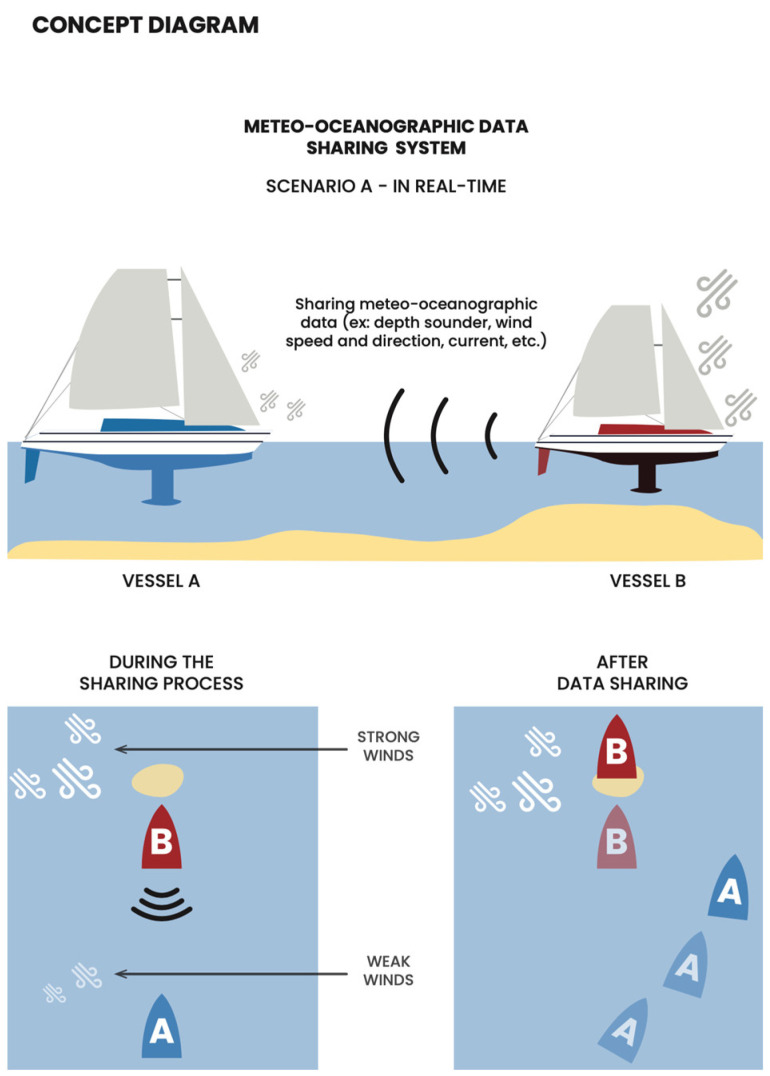
Meteoceanographic data sharing system research concept.

Despite both offshore and coastal waters presenting challenges to navigation, such as limited infrastructure for immediate aid [10], or intense currents and unexpected winds, inland waters pose even more demanding navigation conditions due to shallower depths, narrower channels, intertidal channels, and non-linear tidal propagation [11], demanding precise and anticipatory data on a vessel’s position and the real water depth [10,12] to avoid grounding. Due to these conditions, it is particularly challenging for ships and vessels with drafts higher than the available water height (observed depth) [13] to navigate through shallow navigation channels, such as bars, rivers, and harbors [12,14]. Shallow channels pose a risk of grounding, which can lead to significant delays and potential damage to vessels. To mitigate the aforementioned risk, Figure 1 illustrates a conceptual approach to the data sharing system. This system allows Boat B to share real-time data with Boat A. These data may encompass information regarding the presence of shallow areas, strong winds, or other potential hazards that Boat B has previously encountered. By receiving this information, Boat A is thereby enabled to anticipate and adjust its maneuvers in advance, thus avoiding these dangers and ensuring a safer and more efficient passage.

There are several factors that impact navigation safety, efficiency, and situational awareness within an environment that require sailors to be cautious and anticipate maneuvers in order to avoid hazardous conditions for vessels, and these include the following: (i) severe meteorological conditions, such as storms, heavy rains and strong winds, not only pose direct dangers to navigation but also make it more difficult to accurately perceive water conditions (e.g., currents and tidal heights) [15,16]; therefore, accurate local hydrodynamic and weather forecasting is crucial to mitigate risks, such as grounding; (ii) uneven water flow propagation, which is especially problematic in inland waters, causes non-linear tidal propagation [14,17]; (iii) high expenses from hardware, software, and network components, such as River Information Services (RIS), which are required to obtain real-time and accurate information for nautical charts; (iv) low tide and the inadequate coverage of shallow waters [12,18]; however, updated buoys and lateral markers indicate safe routes [1,18], and depth sounders provide real-time information on water depth under the boat keel [19]; and (v) complex interface designs that delay sailors’ decision-making [20,21,22].

The challenges posed by maritime environments highlight the importance of using and leveraging advanced navigation technologies and systems, such as hybrid satellite–terrestrial communication networks, autonomous systems, and digitalization to enhance navigation safety and efficiency [23,24,25]. Hence, the most accurate and localized meteoceanographic forecasts are necessary to anticipate and manage adverse conditions effectively.

In Portugal, the Ria de Aveiro, a coastal lagoon on the west coast [14,26], exemplifies these challenges. The lagoon’s shallow depths and the non-linear tidal propagation in most navigation channels highlight the need for precise and reliable navigational aids and forecasting tools. Although the system can operate in both coastal and inland contexts, the Ria de Aveiro is particularly important for this research field—maritime navigation studies—as an estuarine inland waterway, a setting that is rarely explored.

To fill the gap in existing information systems capable of sharing and updating real-time navigability data, a comprehensive system for the visualization and sharing of meteorological and oceanographic data is presented in this article, encompassing the following features and benefits: (i) real-time data collection using existing boats sensors allows real-time data to be collected about the local water depth, currents, water temperature, and weather conditions (wind speed and direction); (ii) hydrodynamic and weather forecasting using weather and hydrodynamic models to predict wind and tidal behavior along water channels; and (iii) a low-cost and user-friendly platform for sharing and visualizing real-time and forecasted data.

This novel maritime data sharing system using IoT technologies, specifically designed to enhance navigation in both inland and coastal waters, including offshore environments, uses low-cost electronic components to enable sailors to access and share real-time and deferred oceanographic, meteorological, and vessel data. The distinguishing feature of this system is not solely its competitive pricing, but also the use of local tidal predictions from a dedicated hydrodynamic model that provides real-time navigational information, namely the tidal height at any given point or moment in the inland water plan, and the augmented routes within the Ria de Aveiro (an augmented route contains refers to a navigational path that has been augmented with supplementary, real-time, or dynamic data, such as tide height information, which is of paramount importance for determining navigability conditions). Furthermore, the system enables the sharing of vessel-specific data, including speed over ground, heading, and direction, aggregated with meteorological and oceanographic updates.

The selection of the Ria de Aveiro as a case study underscores its unique characteristics and navigational challenges. Leveraging this technology provides real-time meteorological data, enhancing safety in intricate maritime environments. As we move forward, the integration of Information and Communication Technology (ICT) and emerging technologies like Big Data and Artificial Intelligence will continue to shape the maritime industry, fostering safer and more efficient operations.

The research’s primary objective is to illustrate the system’s potential to enhance maritime navigation safety. A Contextual Design approach [27] is employed to adapt the system to meet the specific needs of sailors, addressing a gap in existing solutions by offering an innovative, cost-effective approach to enhancing navigational safety and efficiency, assisting vessels while navigating. It is expected to contribute to the existing knowledge by presenting a practical solution to the navigational challenges faced in inland and coastal waters.

This article is structured into five sections. After the introduction, the theoretical framework underlying the main research concepts is introduced. The third section presents the latest technological solutions available in the market, and details about the system’s architecture and user interface (UI) developed in this study. The fourth section discusses the results of questionnaires and interviews conducted with users of the solution developed in this study. The fifth section presents a discussion of the results and provides conclusions to the research and on the integration of the maritime data sharing system with community sharing mechanisms.

## 2. Advancements in Maritime Technologies: Enhancing Navigational Safety and Communication

The system presented in this study, which integrates the e-navigation objectives, offers a potential avenue to enhance existing platforms and equipment that facilitate maritime navigation. Indeed, the integration of ICT has led to the development and integration of Big Data and Artificial Intelligence [28,29], which improve the analysis of information about the vessel, such as Automatic Identification System (AIS) logs, meteorological information, and data acquired from different sensors, to share and compare them with data from other companies and entities [30].

Complementarily, the Internet of Things (IoT) [31,32] is a relevant technology and, in recent years, has contributed to assisting the maritime sector with ship maneuvers, port management, and aid and rescue [30,33]. The basis of an IoT system is collecting and sharing information about the electronics installed on the vessels [24,34,35], which can aid the sailors’ decision-making and improve communication between boats.

The rapid advancement of ICT technologies has transformed navigation tools [30,36,37], enabling ship-to-land communication, route visualization, access to route information, and the management of collision risks between vessels, all through a single interface [30]. Notwithstanding the numerous benefits of such technologies for the sailors, there are still two main gaps that must be addressed: (i) human error, since sailors may become over-reliant on the system and disconnected from their environment [38,39,40], and (ii) the timely sharing of information, such as weather and oceanographic forecasts, namely between communities of sailors, which can contribute to safe route planning [41,42,43].

Although many leisure vessels are equipped with useful instruments, improving the communication between sailors and the UI of electronic devices remains essential to ensure distraction-free navigation [44,45]. Displaying these data on advanced onboard systems, such as plotters, Multifunctional Displays (MFDs), and Electronic Chart Displays (ECDISs), provides crew members with continuous access to critical navigational information [46].

Table 1 summarizes the reasons for checking specific data during maritime navigation, and where and how these data are visualized and integrated into the navigation systems:

Consider two sailors, A and B (Figure 1). Sailor B acquires the observed depth data while navigating, and Sailor A is interested in understanding the navigability conditions in the same area where Sailor B was located. To facilitate this, the system retrieves the water depth data from Sailor B and sends it to a server, which then calls an API to obtain both past and future tide heights for the area. Using this information, an algorithm processes each of Sailor B’s data points, calculating the observed depth relevant to Sailor A [12]. By combining this value with Sailor A’s vessel draught, the system is able to display an augmented route. Therefore, both the observed water depth and tide height are critical in this process.

Accessing shared meteo-oceanographic data provided by other vessels’ electronic sensors (such as anemometers for wind direction and speed, and transducers for water depth), and representing it accurately through a UI that adapts the information collected to the navigational context (i.e., environmental factors–sunlight and rain), reduces risks during navigation [8,47]. Consequently, the objective of this research is to explore the access to data shared by vessels and use that to aid other vessels.

Also, deferred acquisition allows for the analysis of data acquired during a voyage and the subsequent optimization of future routes, thereby contributing to overall maritime safety and efficiency [8,12,48]. The sharing of these types of data is also crucial to update or create hydrodynamic predictive models, including input from scientists and experts as well as regular and experienced sailors.

In conclusion, the theoretical framework presented in this paper identifies a significant gap in the existing solutions for real-time navigational support in maritime environments. Current technologies often have high costs and maintenance is complex, making them less accessible and practical for widespread adoption. Consequently, there is a critical need for innovative solutions that are affordable, user-friendly, and robust to enhance maritime safety, in both coastal and inland waters. The proposed system is designed to be affordable, easy to use and robust for navigational safety. It is intended to serve as a new standard in this field, exemplified by the unique challenges and opportunities presented by the Ria de Aveiro.

## 3. Low-Cost Maritime Data-Sharing System Design and Evaluation

This section delves into the systematic process employed to achieve the study’s goals, applying several techniques and methodologies, namely a comparative analysis of available technologies and data sharing systems as well as the UI designed to deliver the data sharing system to the target audience and its architecture.

A core element of this procedure entailed the collation of insights through a targeted survey, which provided indispensable feedback on user needs and preferences. These insights proved invaluable in guiding the development of the low-cost system, ensuring that it met the identified requirements. The integration of user feedback with the technical design allowed for a refined and user-centered approach. This comprehensive exploration not only addressed the complex challenges inherent to maritime operations but also enabled the identification and validation of both functional and design requirements. These requirements were subsequently incorporated into the data-sharing system, ensuring that it is both effective and responsive to real-world needs.

Moreover, it addresses the implementation of a nautical simulation station to test the sharing and visualizing system. The exploration of these techniques provided insights into the strategies and tools used to address the complex challenges inherent to maritime operations and allowed us to identify and validate the functional and design requirements that needed to be incorporated into the system itself and its prototype.

The case study methodology [49,50] was applied, leveraging a phenomenological approach to explore specific contexts within the Aveiro’s lagoon. This methodology allows for a comprehensive examination of societal actions impacting the environment [50], particularly in the realm of maritime navigation. The case study is a common approach in maritime navigation studies, which aims to improve human–machine cooperation and enhance situational awareness and decision-making for sailors [51]. Nonetheless, as the sharing system will be used by humans, it is important to evaluate its usability in an outdoor and maritime context. For that reason, the Contextual Design (CD) approach was used [27], which integrates the design into a participatory and iterative process and analyzes it in a specific context. CD allowed this research to create a centralized UI design process to be integrated into the data sharing system based on the sailors’ needs.

### 3.1. Techniques for Supporting Maritime Information Sharing and Visualization: A Benchmarking Analysis

To inform the design and implementation of the data sharing system in this study, an understanding of current maritime technologies is essential. Therefore, a comparative study, known as benchmarking, was performed. Benchmarking is a useful approach for comparing practices and products developed by competitors, aiming to identify gaps in existing solutions that could be addressed by research [52,53,54].

A benchmarking process was conducted to identify and compare several navigation support systems, including proof of concepts, nautical applications, and equipment such as *chartplotters* and AIS, developed both academically and industrially. Each of these solutions aligns with the objectives outlined for this research:

- Solutions facilitating real-time data sharing and visualization with other users;
- Solutions representing meteoceanographic conditions and navigational information along maritime routes.

This study builds on a previous benchmarking study of mobile nautical applications [55]; however, it was updated to focus on developing the presented low-cost IoT solution. A comparison with commercially certified devices is valuable for understanding the state-of the art in both functionality and UI design. By analyzing these established products, key features that are essential for similar systems can be identified. This comparison ensures that the system not only meets functional requirements but also adheres to UI conventions commonly used in maritime applications. By maintaining these established standards, a fluid user experience can be ensured, allowing navigators to interact with the interface comfortably and effectively as they are already accustomed some of these common practices.

The identification of solutions was carried out by searching across mobile application stores, such as the App Store and Play Store, scholarly databases, such as IEEE Explore, Scopus, and Web of Science, Facebook groups, and a general Google search using terms, such as “data sharing systems to support maritime navigation”, “sharing data from marine navigation devices”, “maritime navigation devices sharing information”, and “NMEA usage to sharing navigation devices data”.

Eighteen technological solutions, including mobile applications and navigation support tools, were thoroughly examined to determine how they can be used to share meteoceanographic data and verify if those services could incorporate important information to improve maritime safety, such as updated signalization data, cartography, and bathymetric data. This research has contributed to improving the evaluation of maritime technologies by offering new perspectives and has offered important insights into the effectiveness, advantages, weaknesses, innovation, and cost-effectiveness of these solutions.

Currently, the maritime community can make use of a variety of tools to access pertinent information that assists in navigation, which can be acquired through (i) sensors situated in the maritime environment that transmit data to a central hub and subsequently disseminate it to sailors [56] or (ii) sensors installed on the navigators’ vessels that allow sailors to share relevant data [57,58]. However, the first type of solutions, such as smart buoys and tide gauges, have significant limitations, namely high costs [58]. On the contrary, solutions that enable sailors to collect and share meteorological data through electronic equipment installed on vessels are gaining prominence. This capability is crucial for aiding early decision-making while navigating maritime routes [57,58,59].

Consequently, it is imperative to comprehend the main technologies available for accessing such data that can help navigators to anticipate their sailing maneuvers towards safe navigation. The solutions found in this analysis are presented in Table 2, delineating the advantages and disadvantages of each approach, according to boat sensor connectivity; access to inland water data (i.e., the data from hydrodynamic models can be accessed in order to determine the height of the tide and to make predictions regarding the hourly sounding at a specific location and time) or hydrodynamic models; user interface design parameters that present the essential vessel data in a systematic and readily accessible layout, ensuring that the navigator can readily obtain the information they require; an evaluation of the user interface in an outdoor context (i.e., limited to projects with functional prototypes available); the possibility of sharing data to a community; free access for all application or systems functionalities; and finally, the cost.

One application was found that was capable of accurately simulating navigational conditions in inland waters, Routinav, although it is limited to a singular context (the Aveiro’s Lagoon). Routinav has access to a sophisticated hydrodynamic model that enables the calculation of tidal heights at any point and time within the inland water plane of Ria de Aveiro. This unique feature enables Routinav to provide precise and reliable information on the observed depth, which is crucial for safe and efficient navigation in such complex environments.

In this regard, services such as Navionics Sonar Map [66] and OpenSeaMap [73] have adopted data sharing based on routes acquired by users to enhance digital nautical charts with bathymetric information. Sharing such data aids in updating crucial information that is essential for navigation. However, validating shared information in both applications and navigation support systems is crucial, as these services lack a robust method to rectify shared data discrepancies. To address this, adjustments such as tidal corrections along the water plan are necessary, utilizing either tidal gauge information or the results of hydrodynamic models.

The analysis of the electronic components and implemented interfaces used supports this research to create a solution that accurately represents and refines information before sharing it with the maritime community. Raspberry Pi emerges as a promising low-cost solution due to its ability to integrate various sensors and devices for maritime data collection, along with its low power consumption, making it ideal for maritime applications.

Among the analyzed products and projects, BBN [62] stands out for its utilization of Raspberry Pi, enabling the aggregation of crucial navigation information into a single interface.

Regarding data representation using a single interface, Saillogger [71] and SailRacer [74] offer several advantages over other applications and services. These two apps synthesize key data to reduce sailors’ cognitive load and streamline the decision-making process. Moreover, in the case of Saillogger, the cost of the equipment is lower than the average for the applications analyzed.

Meanwhile, Savvy Navvy maintains visual coherence in its interface, offering intuitive graphics and detailed application guides. Investigating the e-Paper technologies mentioned in SailRacer [74] may address the limitations encountered during data communication and visualization in maritime contexts.

Throughout this benchmarking, the primary data presented across interfaces of the analyzed systems and applications included water and ground speed, position, current, depth, wind, and vessel heading. Table 2 summarizes an analysis of the key functionalities that needed to be integrated into the maritime sharing system developed. These criteria consider whether the analyzed services connect with boat sensors to minimize implementation costs and ensure visual consistency and ease of use in maritime contexts.

Furthermore, following an exhaustive examination of the available technological solutions and electronic components, it was determined that Raspberry Pi is a highly versatile device with a competitive price point in comparison to the various solutions currently on the market, presently costing around EUR 62. To further enhance the system, a 7” Raspberry Pi touchscreen was also acquired at a cost of around EUR 73, and a case to safeguard the system was acquired at a cost of around EUR 8. Furthermore, the ESP8266 is a cost-effective microcontroller, priced at around EUR 8, with fundamental capabilities for integration into an IoT network. In this regard, the total cost of the system, including the memory card, two heatsinks, and a power cable, amounted to around EUR 164. Based on the findings of this research, the system’s cost is relatively affordable, as it incorporates essential features for navigation in various environments, including inland and coastal waters, which are lacking in other solutions.

The benchmarking study provided valuable insights that were essential to the preparation of the data collection process regarding the crucial data that needed to be shared within the low-cost maritime data sharing system. Moreover, this comparative study contributed to supporting the conceptualization of the maritime data sharing UI.

### 3.2. System’s User Interface (UI)

This section explores the crucial phase of designing and validating the UI elements. An effective UI design that aligns with users’ needs is essential for enhancing the user’s experience and interactions with the system [75,76]. This is particularly important in maritime contexts, where visual clarity and usability are vital to supporting maritime safety, especially during decision-making and risk communication processes.

**Figure 2:**
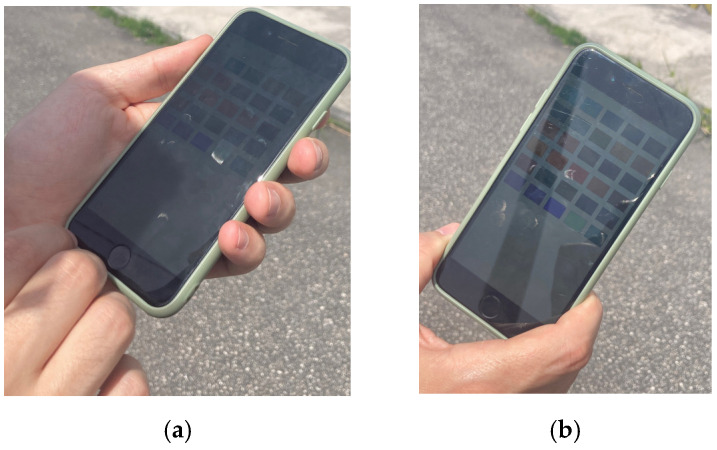
Outdoor color palette validation: (**a**) direct sunlight exposure on the screen during testing; (**b**) the set of colors chosen for evaluation.

**Figure 3:**
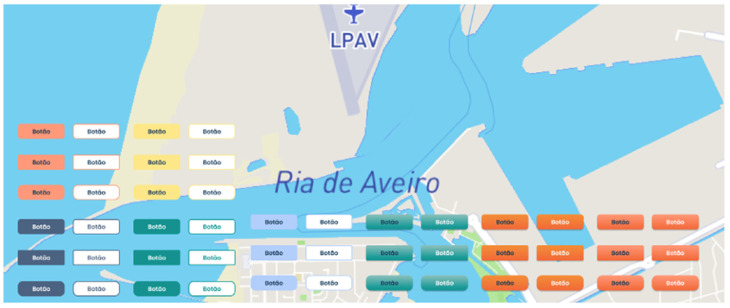
UI with multicolored buttons evaluated in an outdoor context.

The maritime sailing environment (e.g., direct sunlight) can lead to Situational Visual Impairment (SVI), so it is essential to validate interfaces and functional requirements with the mariners who will be using them. Gathering feedback from those involved in maritime activities ensures that systems are aligned with user needs. Addressing SVI is critical in maritime operations where environmental factors affect visual perception and information processing [77,78]. Therefore, a set of colors were observed and assessed under direct sunlight (Figure 2 and Figure 3). To evaluate the system’s final performance, an iPhone SE 2020 [79] with 625 nits was used.

The evaluation showed that navy/dark blue and orange are the most legible colors in outdoor environments, making them ideal to be as the primary colors for the UI. In addition, the typography should be in the darkest shades possible to create a strong contrast and make the text legible, ensuring access to key actions within the UI while navigating in all maritime conditions.

With the color palette and benchmarking analysis in place, it was easier to conceptualize ideas for integration with existing data-sharing systems to design the most optimized UI solution. Solutions such as a Navionics Sonar Map, Sea.AI, OpenSeaMap, SailRacer, and Savvy Navvy [65,66,67,80] aided in representing maritime data in a UI, particularly by using larger numbers and concise textual descriptions to indicate the type of data being presented. Furthermore, the Waze application [81], originally designed for road navigation, influenced the maritime data sharing UI design by introducing crucial features, such as user identification through customizable avatars, a directional arrow indicator for the user’s vehicle, a hazard button for reporting traffic or road hazards, and summarized data about road navigation.

**Figure 4:**
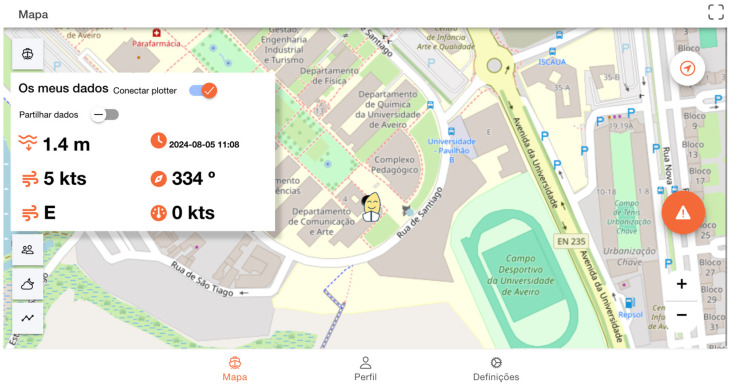
The system shares real-time data, such as water depth, wind speed and direction, course, and speed over ground (SOG) with the community.

The interface was developed to be able to synthesize data received from the user’s NMEA network (Figure 4). The user can choose whether to share these data with others (Figure 4–in Portuguese, “*Partilhar dados*”). The technical process of data sharing between vessels and how sensors contribute to this process can be better understood in the following section.

**Figure 5:**
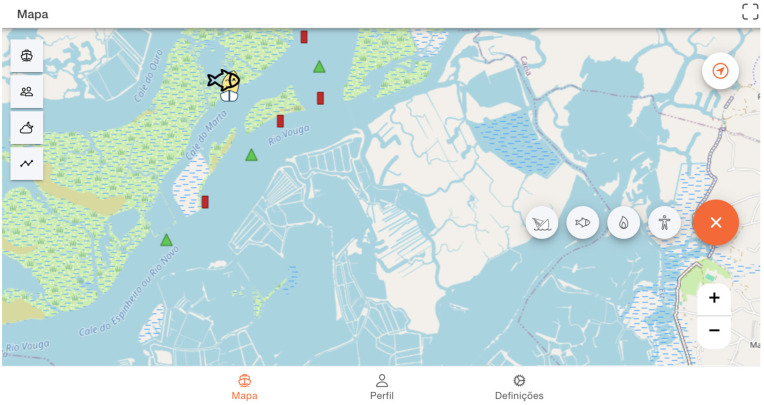
Meteo-oceanographic data-sharing system UI buttons. The orange button on the bottom represents the emergency button to report accidents and other complementary information to the maritime environment.

Additionally, an emergency button was included to report incidents, such as shipwrecks, collisions, groundings, and men overboard. These reports are automatically displayed to all members using the data sharing system, similarly to Waze (Figure 5).

**Figure 6:**
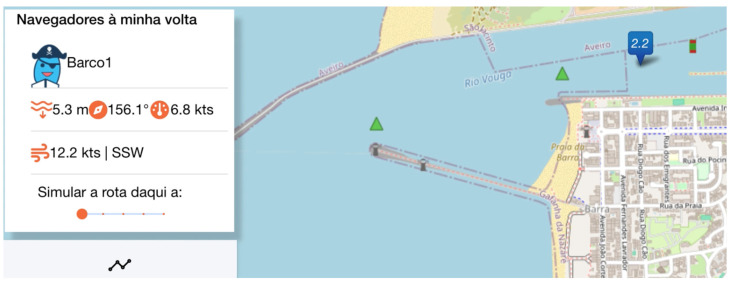
Meteo-oceanographic data sharing system UI with two sections: data connected to the NMEA boat’s network and the information shared by users in the local area.

**Figure 7:**
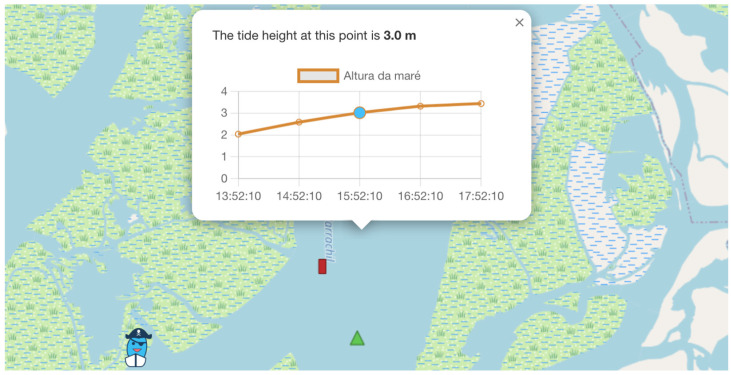
Tidal height from a specific point in the Ria de Aveiro.

The feature “Sailors Around You” (in Portuguese “*Navegadores à minha volta*”) enables the users to monitor nearby boats identified by their avatar, boat name, and shared meteorological and oceanographic data, such as water depth and wind (Figure 6). Additionally, users can receive real-time tidal height information about any selected area by clicking on any given point in the main map (Figure 7).

The UI design presented in this section was based on the features of existing technological solutions in academia and industry (as described in the Benchmarking subsection), IMO guidelines [82], and the Nielsen and Norman UI design heuristics [83,84]. In addition, situational visual limitations [77,85] have been considered, in particular, the impact of direct sunlight on navigational challenges.

### 3.3. Maritime Real-Time Data-Sharing: The NMEA Network Simulator and System’s Architecture

Before delving into the development of the low-cost IoT solution, it was essential to build a small simulator for a maritime usage scenario. This simulator station was equipped with a set of nautical electronic instruments to simulate an actual boat scenario.

Several solutions examined in the benchmarking [35,51,58,61,62] revealed the possibility of accessing data obtained by existing sensors on nautical vessels and sharing them via the NMEA (National Marine Electronics Association) protocol [86,87]. NMEA was introduced in the 1980s to consolidate and transmit all data from various maritime electronic instruments through a single electronic device [86].

In the NMEA network, there are two types of networks: the NMEA 2000 network and the NMEA 0183 network. The main difference is that in NMEA 0183 each sensor is connected to the network, while in NMEA 2000, the sensors are grouped into a single connector that simplifies the connection with the rest of the electronic sensors. This communication protocol stands out in this study since it serves as the foundation for connecting the data-sharing system to a significant portion of the instrumentation found on a vessel. This is a crucial protocol, responsible for connecting the boat’s sensors to a specific network to consolidate all of the data obtained.

For this station a B&G Vulcan 7 MFD [88] was purchased, which includes GPS, Radar, and an AIS, primarily used as an aggregator of sensor data connected to the NMEA 2000 network through Wi-Fi, which allows users to receive and process these data on an external interface. The wind (B&G WS310 [89]), the water depth, the speed, and the air temperature sensors (Airmar B&G DST 800 [90]) were also integrated into this simulation station.

**Figure 8:**
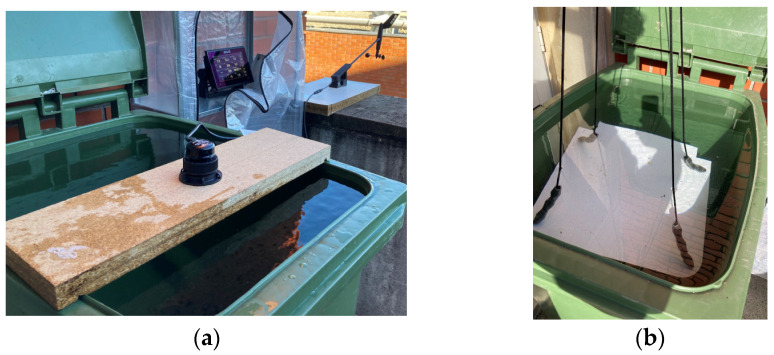
The NMEA network simulator: (**a**) It is possible to observe the depth sounder support by a wooden board, the B&G plotter inside the storage shelves, and, on the right side, the wind sensor. (**b**) The water tank contains three 30 g weights at each end of the K-line and four cables to raise and lower it, simulating the height of the tide.

The depth sounder presented a significant challenge in the simulation station, since accurate readings required a minimum depth of approximately 1 m and, preferably, it should be inside water. To tackle this issue, a trash bin was used to fill it with water and the sounder was placed on a wooden platform (Figure 8a). A K-line with adjustable weights was used to raise and lower the false bottom to simulate variable bottom conditions (Figure 8b), such as those encountered when navigating inland waterways.

**Figure 9:**
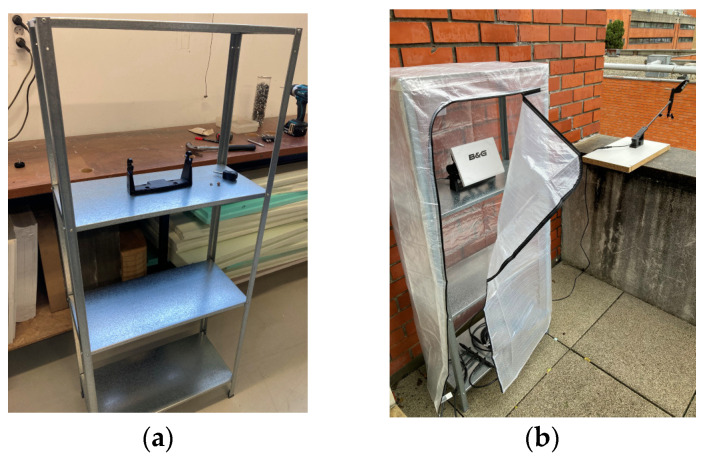
The simulator montage: (**a**) the storage shelf prepared to position the plotter on the highest available surface; (**b**) the outside plotter and wind sensor installation.

To ensure that the GPS signal was received properly, the plotter had to be placed on a storage shelf (Figure 9a), while the anemometer was mounted on a robust wooden platform to ensure stability in case of strong winds. This setup was installed on an outdoor balcony at the university department to simulate real-world conditions and obtain a preliminary simulation of a real usage scenario (Figure 9b).

The establishment of an NMEA network was facilitated by this simulated maritime data-sharing station. Accessing NMEA data became feasible through a two-step process: (i) programming the ESP8266 using an Arduino script; (ii) and then using a Python script via the serial port on the Raspberry Pi. This Python script effectively read and processed the NMEA data, rendering it into a readable format for the API.

Raspberry Pi was chosen due to its versatility and processing power, which were identified during the benchmarking process. It has been widely used in previous projects and is suitable for small-scale projects. The computer is highly adaptable and offers a robust platform for developing and deploying various applications, including those requiring real-time data processing and transmission, such as this maritime data sharing system. The cost-effectiveness and availability of this solution also make it an attractive option.

Furthermore, the ESP8266 Wi-Fi module [91,92,93] was chosen for its ability to establish a dependable serial connection with the Raspberry Pi [94,95], enabling the retrieval of NMEA data from the plotter. Its compatibility with serial communication protocols and compact form factor makes it a suitable component for integration into the system architecture. This hardware system has the potential to develop a low-cost solution that does not compromise functionality or performance.

The Raspberry Pi and ESP8266 combination was leveraged to achieve the necessary capabilities for data transmission [95], enabling a seamless integration into maritime environments with varying levels of instrumentation. The strategic pairing of the Raspberry Pi and ESP8266 empowers the maritime sharing system to efficiently retrieve NMEA data, contributing to the overall effectiveness of maritime operations.

A Raspberry Pi 3 A+ model was initially used to implement a dual Wi-Fi functionality. However, this setup faced connectivity issues as the Raspberry Pi kept disconnecting from the plotter’s Wi-Fi network and connecting to a domestic network after a few seconds. This affected data access through a REST API. To address this issue, an ESP8266 device was introduced to enable a serial port connection between the Raspberry Pi [94] and NMEA network data. The data were then transmitted to the local network where the Raspberry Pi was connected.

It was possible to analyze the sharing of NMEA data from the Raspberry Pi 3 Model A+ to the Firebase. This test showed that the Raspberry Pi 3 model A+ was not suitable due to its limited processing power and RAM. Upgrading to the Raspberry Pi 3 B+ model resulted in a slight improvement, but performance issues persisted. In conclusion, the Raspberry Pi 4 B+ with 4 GB RAM demonstrated a higher information processing speed and responsiveness, particularly in handling front-end and back-end tasks.

The final solution used a Raspberry Pi 4 B+ with an ESP8266 serial connection to retrieve NMEA data. This low-cost solution enables vessels with some nautical instrumentation to share data. Initially, a Python script was used for data storage and Firebase transmission. Despite the delayed responses resulting from API requests, the Vue.js website effectively utilizes a Web API Serial to read the connection values from the ESP8266 plotter. VUE.js [96] was used to display, process and store the received data in the Firebase database.

**Figure 10:**
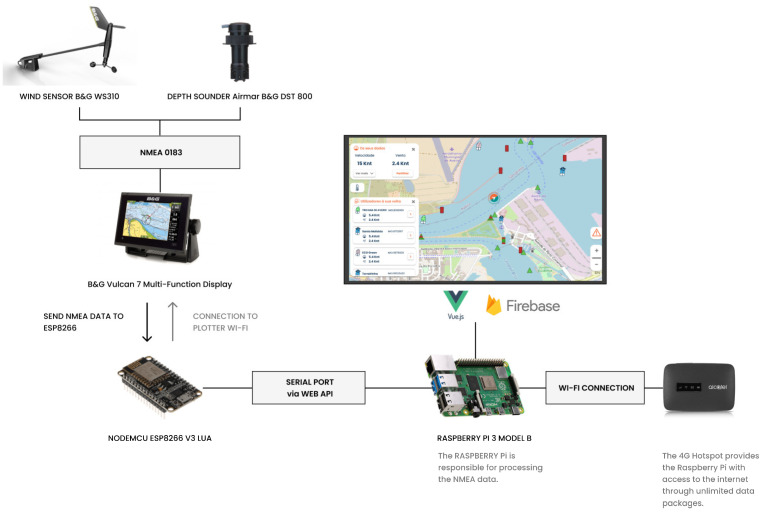
The final architecture proposed for the maritime data-sharing system.

The final architecture of the data-sharing system (Figure 10) maintained the previously proposed hardware setup, but the Flask API functionality was removed because it was not possible to send requests from the Firebase host to the Raspberry Pi API. To address this, the Firebase host was kept, and the front-end code (VUE.js) was modified so that the ESP8266 could read the data directly, bypassing the need for the Flask Python API. This change reduced latency and improved the Raspberry Pi’s efficiency in displaying and storing data.

As shown in Figure 10, the Raspberry Pi connects to a Wi-Fi network (i.e., 4 G hotspot) available on the boat, which not only communicates with the VUE.js interface to receive data from the Firebase database but also updates the database in real-time. This connection ensures that the shared data are continuously updated in real-time, allowing for accurate and timely information sharing.

## 4. Survey and in-Context Interviews

In marine research, it is essential to understand the characteristics of the sample population. Therefore, this research takes a user-centered approach to UI design and involves sailors throughout the entire research process. This study examines the demographics, license status and vessel instrumentation of marine enthusiasts. The results provide valuable insights into the sample population and the potential for data sharing between vessels, which is the main focus of this study.

This section presents findings from surveys and interviews (approved by the General Data Protection Regulation (GDPR) office from the University of Aveiro (see Appendix A)) with sailors from nautical associations within the Ria de Aveiro to assess the low-cost maritime data sharing system developed and presented in previous sections. This approach allowed us to observe and question individuals with a specific level of experience in maritime navigation, as they hold pertinent knowledge regarding the phenomenon under study. Participants were part of the maritime and nautical industry or the fishing and recreational navigation sectors. The sample selection method employed was a non-probabilistic intentional sampling method [97,98,99].

In the initial stage, an online survey was conducted with a total of 22 questions (N = 22), which was hosted on the University of Aveiro servers. A subset of these participants was able to be interviewed, resulting in a total of 11 participants (N = 11). Although it is not possible to confirm the number of sailors in the Ria de Aveiro from official documents, an effort was made to involve experienced sailors belonging to the region’s nautical associations in order to ensure the best possible representation of the study’s population.

### 4.1. End-User Validation of the Meteo-Oceanographic Data-Sharing Concept: Survey and Interview Results

Questionnaires and semi-structured interviews were the chosen methods to support these evaluation sessions, since they could enrich the research [98]:

- The interviews allowed the sailors to identify relevant data to be shared within the system, and facilitated an open a debate, resulting in a set of UI designs that could be integrated into the maritime data sharing system;
- The questionnaires aimed to identify participants’ characteristics (e.g., navigator’s license, sailing frequency, onboard electronics) and opinions about the functional requirements of the maritime data-sharing system.

**Figure 11:**
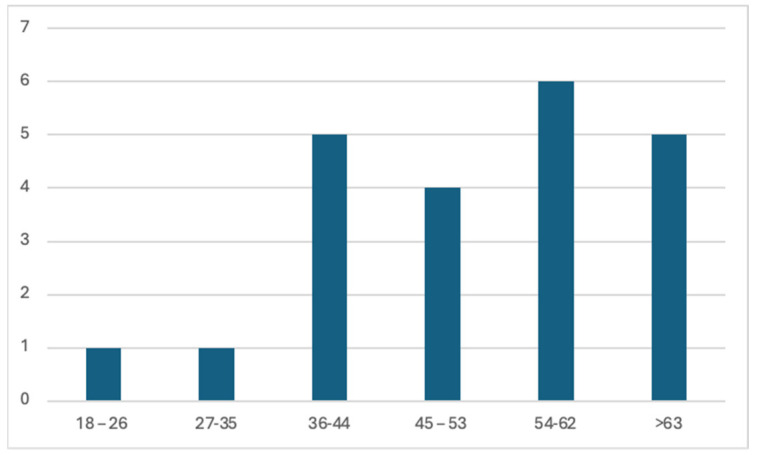
Participants’ age range.

The participants in this study spanned a diverse age range (Figure 11), reflecting the multifaceted nature of the maritime community. The age distribution underscores the inclusivity of the study sample, encompassing both seasoned sailors (e.g., leisure) and those embarking on more complex maritime journeys. However, it can be seen that there is a greater concentration of people aged over 36. The age diversity enriches this research, allowing us to understand the challenges and experiences faced by sailors across different life stages.

**Figure 12:**
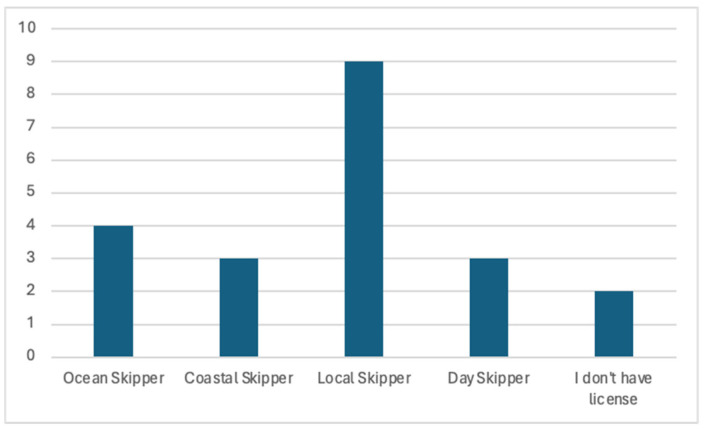
Participants’ boat credentials.

Figure 12 illustrates their licensing credentials. The chart displays the licenses held by the participants: two participants (n = 2) did not have a license, four (n = 4) held an ocean skipper license, three (n = 3) held a coastal skipper license, nine (n = 9) held a local skipper license, and three (n = 3) held a day skipper license. These licenses provided this study with information about their maritime ability. Each category represents a distinct level of nautical competence, acquired through training, experience, and adherence to safety protocols. The diversity of licenses reflects the varied roles these individuals play within the maritime ecosystem.

The study spreads to vessel ownership and electronic instrumentation. These aspects significantly influence the operational capabilities and safety practices of sailors. It is important to note that out of these participants, two did not have their own vessel (n = 2), eight participants (n = 8) did not have electronic instrumentation on their vessel, and only nine participants had instrumentation (n = 9).

The dichotomy between vessel owners and non-owners sheds light on resource access, responsibilities, and the ability to engage in independent journeys. While some participants rely on shared vessels or charters, others enjoy the autonomy of their craft. Notably, those participants who do not possess their own vessels are, in fact, maritime professionals within the merchant navy.

**Figure 13:**
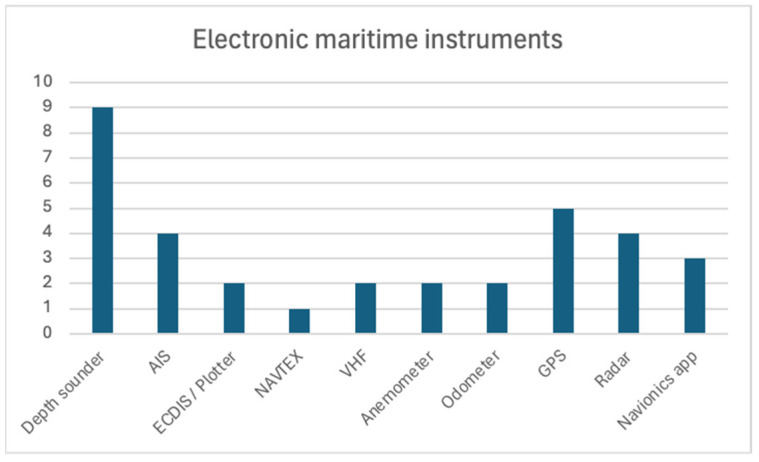
Most used maritime electronic instruments installed on participants’ boats.

Electronic instrumentation—such as GPS, radar, and communication devices—plays a pivotal role in modern navigation. This analysis explores the electronic instruments most employed by the participants—participants had the option to select multiple instruments. The survey revealed the most used electronic instruments among the participants: depth sonder (n = 9), GPS (n = 5), radar (n = 4), and AIS (n = 4). The following chart (Figure 13) emerged from their responses.

The prevalence of electronic instruments among these participants underscores their adaptability to technological advancements. However, the presence of non-instrumented vessels highlights the need for continued education and awareness regarding safety practices.

Nonetheless, some participants expressed concerns about the limitations of existing instruments, particularly for accessing information during maritime journeys. These insights highlight the need to address usability issues and technological obsolescence (n = 5) to improve safety and efficiency in maritime operations.

**Figure 14:**
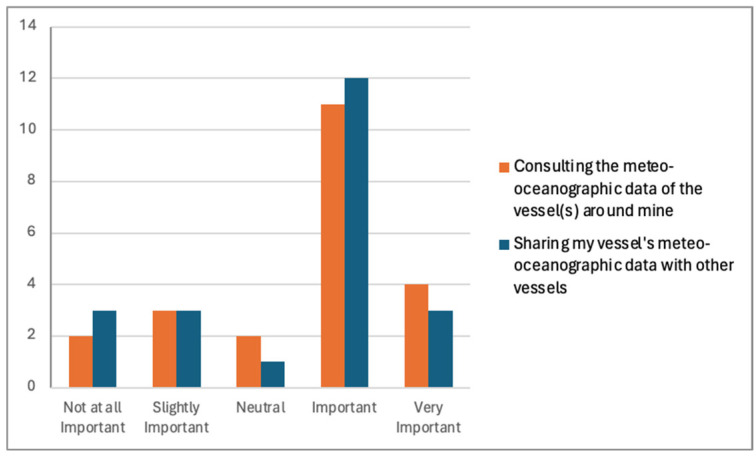
Different opinions about access and sharing meteoceanographic data within an online community.

Figure 14 displays the feedback that highlights the significance of sharing meteorological and oceanographic data between vessels. Upon a closer examination of the data, it is evident that most participants believe that accessing data is crucial, since eleven participants considered it important and an additional four rated it as very important (n = 15). Similarly, when it comes to sharing data, twelve participants highlighted its importance, while three deemed it very important (n = 15). The willingness of participants to engage in data sharing reflects a collective understanding of the importance of collaboration and information exchange in ensuring maritime safety and navigation.

These findings underscore the critical role that real-time data access and sharing play in enhancing operational safety and decision-making efficiency in maritime settings, where conditions can fluctuate rapidly.

Additionally, the identification of key data parameters for sharing highlighted the specific needs and priorities of maritime stakeholders. Participants highlighted the importance of specific data points, such as wind direction, speed, vessel position, and depth soundings, for the safe navigation of coastal and inland waters.

**Figure 15:**
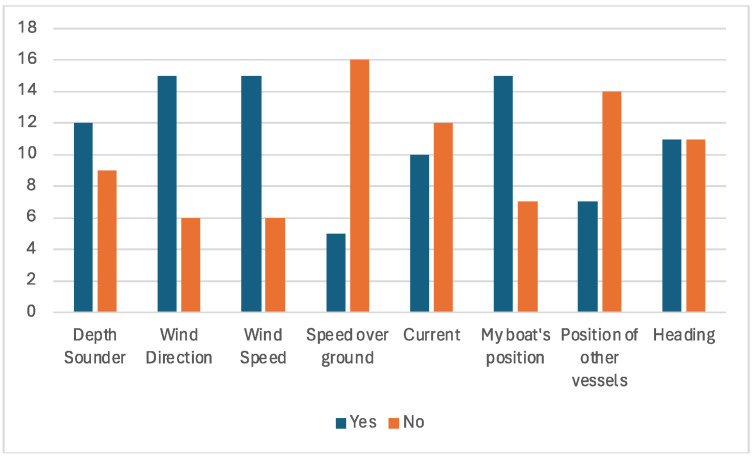
Coastal navigation data that should be shared.

In coastal waters (Figure 15), the most important data to communicate with the data sharing system mentioned were wind direction (eleven considered to be important and four that considered very important–n = 15), wind speed (twelve considered to be important and three considered very important–n = 15), and vessel position (n = 15). Following closely were the depth sounder (n = 12), course (n = 11), and current.

**Figure 16:**
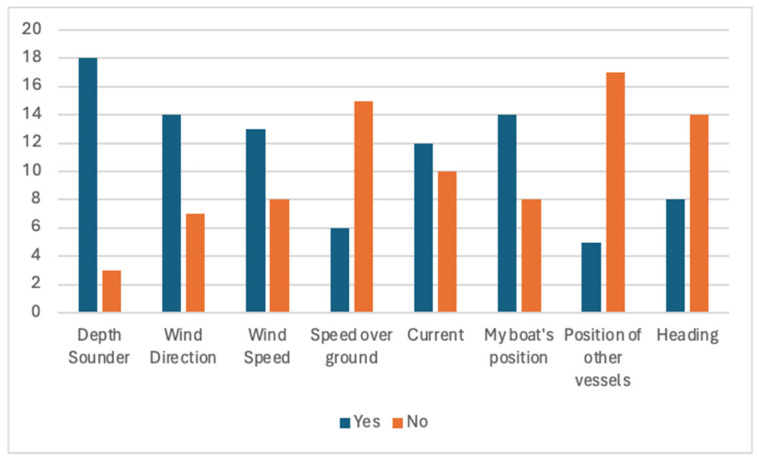
Inland water navigation data that should be shared.

To conclude, inland waters (Figure 16) depth sounder (n = 18), wind direction (n = 14), vessel position (n = 14), and wind speed (n = 13) are the most essential data to share and access.

It is important to emphasize that all these data are integrated into the data sharing system proposed in this article and can be shared between sailors. The concept of sharing data is central to this study since it involves a network of vessels exchanging important information, such as weather updates, navigational insights, safety protocols, and real-time observations.

Sailors are empowered to collectively improve the maritime experience by fostering a culture of openness and collaboration. The potential benefits extend beyond individual vessels to the broader maritime community.

In summary, these results illuminate the maritime landscape, emphasizing the importance of understanding the research population, recognizing licensing diversity, and envisioning a connected maritime community. The survey results emphasize the significance of considering the needs and preferences of maritime stakeholders when developing data sharing systems. By addressing usability concerns and technological limitations, and prioritizing essential data parameters, such systems can enhance safety, efficiency, and collaboration within the maritime community. These findings provide valuable insights for the design and implementation of future maritime data sharing initiatives.

The following section presents the conceptualization of the UI for the data and visualization of the data-sharing system, detailing both the design process and the validation of the color palette for outdoor use.

### 4.2. Outdoor Color and UI Interaction Validation

**Figure 17:**
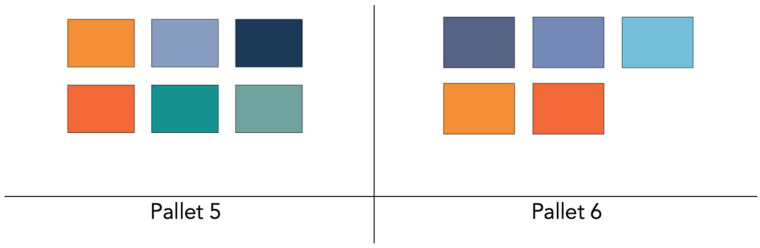
The most predominant palette choices by participants.

Based on the color palette initially analyzed under the influence of direct sunlight, the participants checked which colors they thought would be most suitable for the system. During the interviews with participants (N = 11), six participants (n = 6) chose palette 6 (Figure 17) for the data-sharing system, while the remaining three (n = 3) chose palette 5 (Figure 17). Only two individuals (n = 2) were undecided between palettes 5 and 6. These colors will be incorporated not only into the visual elements of the interface but also will form the basis for the map style and user icons registered in the system.

Thus, palette number 6 was chosen to initiate the prototyping and harmonization of the data-sharing system’s design. These colors will be incorporated not only into the visual elements of the interface but also will form the basis of the map style and user icons used in the system.

Furthermore, this evaluation provided insights into interface design preferences and necessary data to be shared through the maritime data sharing system. In line with the functional requirements outlined by users in the previous section and based on the presented interfaces, most participants (N = 11) preferred the interface shown previously in Figure 4.

This evaluation provided insights concerning interface design preferences and necessary data to share in the maritime system, in line with the functional requirements outlined by users in the previous section and based on the presented interfaces.

Participant 6 commented that the interface effectively groups all necessary information and allows for easy access to supplementary trip information. Participant 3 emphasized the importance of the information not obstructing their route, while Participant 4 suggested that the interface should include symbols or icons for more direct access to information. When discussing data sharing in an anonymous or public format within this interface, Participant 7 emphasized the importance of public sharing to prevent the spread of ‘fake news’ or false content.

It is worth noting that some participants also contributed to enhancing the interface, based on solutions identified earlier on this article (Benchmarking section). Participant 3 suggested, “Having the height of bridges present in the Ria”; Participant 11 proposed that “The interface should display depth as the first data point, considering that this system will be used in inland water scenarios”; Participant 8 recommended that “I should be able to observe other users around me with simpler and more direct information, without having to click on multiple buttons”; Participant 6 suggested that “I should have the functionality to click on areas in the Ria that allow me to obtain tidal height information.”

In addition, involving stakeholders from the maritime community ensures that the developed systems are not only technologically robust but also practical and usable in real-world scenarios. By seeking input from skippers, captains, sailors, and fishermen, valuable perspectives on the challenges and requirements inherent in maritime activities were gained, enabling to effectively tailor the solutions to address sailors’ specific needs.

In conclusion, the findings highlight the importance of adopting a user-centered approach in designing and developing maritime data sharing systems. By involving end-users throughout the process, systems that enhance situational awareness can be created, improving decision-making, and ultimately contributing to safer and more efficient maritime operations. This approach ensures that the resulting systems are technically sound and responsive to the needs of those who rely on them at sea. It is collaborative and iterative, promoting effective communication and problem-solving.

## 5. Discussion

The results obtained from this research reflect the needs and preferences of sailors concerning data sharing and the use of electronic instruments on their vessels. Participants showed interest in sharing and accessing meteorological and oceanographic information, acknowledging its significance for maritime safety. The identification of important data to be shared, in both coastal and inland waters, emphasizes the significance of this information for sailors and future research within the field.

Understanding tidal movements is crucial for anticipating and mitigating navigational risks, especially in areas such as the Ria de Aveiro in Portugal. Water levels and tidal movements must be carefully monitored. To address these challenges, it is essential to leverage technology. Real-time access to meteorological and oceanographic data can enhance navigational safety by providing sailors with updated information on weather and water levels conditions. Integrating such technology into maritime data sharing systems can help sailors make more informed decisions, reducing the risk of grounding and maritime accidents in intricate environments such as the Ria de Aveiro (Aveiro’s lagoon).

With the interviews and survey, it was possible to emphasize the importance of the data sharing and access to meteorological and oceanographic information, especially in coastal and inland waters where navigation can be challenging. Also, the results of this survey allowed us to understand the importance of human participation in designing a visually appealing and usable UI, especially in contexts where presenting critical information can make a real difference between life and death. It is crucial to consider the insights obtained from individuals who navigate complex situations, as their firsthand experiences often reveal nuanced challenges that may not be immediately apparent. The results about the UI of the maritime data sharing system include opinions from the sample on the best choice of UI for integration into a maritime data sharing system.

The results of this study provide valuable insights for the development of maritime data-sharing systems, highlighting the importance of accessible and reliable solutions that are tailored to user needs. Further research could explore these findings to improve existing technologies and develop new approaches to enhance maritime safety and efficiency.

## 6. Conclusions

With the rapid development of data monitoring sensors, the maritime industry has been able to generate information from different sources, such as vessel movements, every day. The accumulation of substantial amounts of maritime information is creating novel opportunities to stimulate innovation and facilitate increased operational efficiencies throughout the maritime domain. The shipping industry is recognizing the value of data as a means of enhancing decision-making processes, which assist in the anticipation, comprehension, and enhancement of business operations [100,101].

The significance of human life in maritime navigation cannot be overstated, particularly in coastal and inland waters where inadequate signaling and non-linear tidal variations can have a profound impact on vessel circulation and maritime tourism. While technological advancements continue to evolve, it is essential to focus on solutions that address the safety and needs of human life. The proposed low-cost data-sharing system represents a step towards this goal, providing a practical solution that enhances maritime security and operational efficiency.

While technology continues to rapidly improve, it is important to note that creating new technological solutions for the sea or ocean alone is insufficient. It is essential to focus on the development of technologies that address the needs and safety of human life. Human life is the most vulnerable factor when it comes to maritime navigation in coastal and, especially, inland waters.

Although the market already offers several technologies that can be used in the maritime context, an expensive investment is still required, and there is also a lack of tidal height information, specifically for inland waters were the progression of the tides in non-linear. Hence, it is important to stress importance of understanding the key technologies available for accessing these data, and the gaps we need to fill. The new maritime data-sharing system presented in this study tackles these issues since it is a low-cost solution, which benefits from the users’ real-time inputs and from a hydrodynamic model that simulates the tidal height for any given point or time within an inland water scenario. Moreover, by utilizing the NMEA data communication network, this system enables the collection and sharing of critical sensor data, aiding navigational decision-making and enhancing oceanic forecasting models.

Furthermore, this study has demonstrated the feasibility of implementing a low-cost system that can significantly contribute to saving human lives. With the ongoing evolution of technology and the integration of AI and Big Data, these advancements can be further enhanced. By combining the insights from this study with theoretical frameworks and comparative studies (e.g., benchmarking), more effective solutions can be developed to improve maritime safety and efficiency in multiple navigational contexts.

This study has highlighted significant advancements in the development, testing, and modeling of maritime information sensing. The focus areas include large-scale maritime navigation multi-sensor tracking, integration and monitoring meteorological and ocean data fusion, analysis and visualization, forecasting, planning, and decision-making with maritime Big Data.

Hence, this study underscores the importance of combining technological advancements with thorough market research and user engagement. By integrating Big Data and IoT with low-cost meteorological and ocean data sharing systems, the maritime industry can develop more effective solutions to improve safety and efficiency across various navigational contexts. The continued evolution of these technologies holds great potential for saving human lives and enhancing the overall maritime experience.

### Limitations and Future Work

The issues of cybersecurity and the absence of communication signals are aspects that must be addressed in the near future. At this stage of the research, the primary focus was on validating the usefulness of the shared meteoceanographic data, as well as the overall system and user interface (UI) with navigators. Ensuring that the system effectively provides valuable real-time information to sailors was the key objective in this phase, in order to maximize security and prevent maritime accidents. Nevertheless, future work will focus on enhancing the system’s robustness by addressing communication challenges, such as the need for satellite connectivity in areas with limited LTE signals, and mitigating potential cybersecurity vulnerabilities to ensure safe and secure data transmissions between vessels.

The maritime data sharing system presented in this paper is currently undergoing real-world testing in the Ria de Aveiro. These evaluations aim to validate the system’s functionality in a practical maritime environment, specifically focusing on the performance of the sensors and the effectiveness of the data sharing mechanism between vessels using the hardware setup. By testing the system in actual navigation scenarios, we can assess its reliability, accuracy, and overall usability for sailors. This real-world evaluation is a crucial step in refining and optimizing the system to ensure it meets the specific needs of navigators to improve safety and enhance user satisfaction. The iterative feedback gathered from these consequent stages of interaction with the Ria’s sailors were, and will continue to be, used to enhance both the technological aspects and the UI, ensuring that the system is robust and fully capable of supporting navigation in inland and coastal waters.

Additionally, it is important to emphasize that the focus of this work is not to compete with industrial-grade products, but rather to present a proof of concept for a low-cost solution. The affordability of the prototype enables a broader accessibility and ease of use, particularly for smaller vessels operating in inland waters. This makes the system more practical and accessible for a wider range of users.

## Figures and Tables

**Table 1:** Overview of data shared to provide an understanding of the: data types, where the data are visualized, and the primary reasons for sharing the values. This table summarizes a structure to identify the relevant types of information based on the boat’s current situation.

What Is Shared	Where This Data Is Visualized	Primary Reason for Sharing This Data
Wind speed	MFD, plotter, ECDIS or a small analogic display	Allows sailors to adjust their routes and navigation strategies to optimize vessel performance; Aids in preventing dangerous situations such as vessel drifting by anticipating weather and wind changes.
Wind direction	MFD, plotter, ECDIS or a small analogic display	
Water depth (observed depth)	MFD, plotter, or a small analogic display embedded in the boats	Crucial for determining route viability and avoiding shallow or no-go areas; Enables sailors to identify navigable channels to avoid grounding; Ensures safe navigation, especially in unfamiliar areas; Real-time depth data allow for immediate route adjustments, enhancing navigational safety.
Tide Height	MFD, plotter, tide forecast services (e.g., websites and mobile apps) or tide tables	Tidal heights influence the extent of intertidal zones, which are crucial habitats for various marine species. By understanding tidal patterns, vessels can plan their routes to take advantage of favorable tidal currents, reducing travel time and fuel consumption.
Non-meteo-oceanographic data		
Speed over ground (SOG)	MFD, plotter, ECDIS or analogical display	Allows for adjustments in voyage planning and enables real-time decision-making about speed adjustments to maintain safety.
Heading	MFD, (using GPS and Compass), Plotter, ECDIS, AIS, or analog compass	Correcting the heading in real-time to deal with currents and winds that may drift the vessel off its intended course.
Course	MFD (using GPS and compass), plotter, ECDIS or analog display	Confirming that the defined route is being followed accurately, facilitating efficiency and promoting safe navigation.

**Table 2:** Presentation of a systematized comparison of the systems and applications that were subjected to analysis.

Criteria	Boat Sensor Connectivity	Inland Water Data	Friendly UI Design	SVI-Friendly Interface	Community Data-Sharing	Free Software Access	Price in Euros
	Data sharing and visualization system solutions						
Profumo [58]	✓						N/A
Chartplotter (e.g., Simrad GO7 XSR with Basemap) [60]	✓		✓	✓	✓		872.32 EUR
Dynamo [35]	✓		✓		✓		N/A
Mitchell’s project [61]	✓					✓	226.25 EUR
Bareboat Necessities (BBN) [62]	✓		✓			✓	348.33 EUR
Raspberry Pi on boat project [63]	✓					✓	138.91 EUR
Le Diouris project [64]	✓					✓	344.80 EUR
SEA.AI [65]	✓		✓	✓		✓	9999.00 EUR
	Nautical applications to support navigation						
Navionics Boating App [66]	✓						230.68 EUR
AIS-weather App [59]	✓	✓					N/A
Savvy Navy [67]							Free
SmartBoat [68]	✓						N/A
Wärtsilä iSailor [69]	✓						Free
SailRacer [70]	✓						Free
Routinav [12]		✓					N/A
Saillogger [71]	✓						App is Free, but requires a specific hardware that costs 250 EUR–350 EUR
Nebo [72]							183.66 EUR
PredictWind: DataHub [9]	✓		✓	✓	✓		App requires a subscription to access to the main functionalities, and specific hardware that costs 279 EUR
Maritime data sharing system developed by the authors	✓	✓	✓	✓	✓	✓	164.25 EUR

## Data Availability

The data supporting the findings of this study are available from the corresponding author upon reasonable request. However, a detailed description of the datasets analyzed and generated during this study, including links to publicly archived datasets, can be accessed through the MDPI Research Data Policies.
